# Videos in short video sharing platforms as a source of information on bipolar disorder: a cross-sectional content analysis study

**DOI:** 10.3389/fpubh.2025.1627885

**Published:** 2025-10-28

**Authors:** Xin Qi, Qi Lu, Bo Li, Sha Liu, Lei Zhang, Runwu Xiang, Qilong Wang, Dongrong Zhao

**Affiliations:** ^1^Gansu University of Chinese Medicine, Lanzhou, Gansu, China; ^2^Department of Psychiatry, Gansu Provincial People's Hospital, Lanzhou, Gansu, China; ^3^Yan'an University, Xi’an, Shaanxi, China; ^4^Qiqihar Medical College, Qiqihar, Heilongjiang, China

**Keywords:** bipolar disorder, short videos, health information, quality and reliability assessment, content analysis

## Abstract

**Background:**

Bipolar disorder is a prevalent mental health issue characterized by recurrent episodes of mania and depression, significantly impacting patients’ quality of life. With the rise of short video sharing platforms, there is an urgent need to evaluate the quality and reliability of the medical information disseminated regarding this disorder.

**Objective:**

This study aimed to assess the quality and reliability of videos related to bipolar disorder available on popular Chinese short video platforms, including TikTok, Kwai, Bilibili, WeChat, Xiaohongshu, and Baidu.

**Methods:**

A cross-sectional content analysis was conducted in May 2025, using keywords related to bipolar disorder to retrieve relevant videos from selected platforms. The quality of the videos was evaluated using multiple standardized assessment tools, including the JAMA Benchmarking Criteria, GQS, modified DISCERN, PEMAT, and HONCODE.

**Results:**

Significant differences in video quality and audience engagement metrics were observed across platforms. TikTok and Kwai had higher quality scores, while WeChat resulted in more comments. Most videos were created by medical professionals, although independent users also contributed content. Overall, video quality was inconsistent and not necessarily correlated with engagement metrics, highlighting the necessity for improved standards in disseminating health-related information on social media.

**Conclusion:**

On Chinese short video platforms, clinical practitioners are the main creators of bipolar disorder-related content, but their scientific nature, production quality, and information transparency still need to be improved. It is suggested to improve the platform management, creator training, and algorithm optimization, so as to promote the improvement of public mental health literacy.

## Introduction

Bipolar disorder is one of the more prevalent types of mental disorder, and its symptoms can be classified according to severity into bipolar I (experiencing at least one manic episode) and bipolar II (experiencing at least one hypomanic episode and at least one major depressive episode) as defined by the *Diagnostic and Statistical Manual of Mental Disorders, Fifth Edition* (DSM-5) (1). Most patients typically manifest varying degrees of depression or mania, frequently accompanied by co-occurring psychiatric disorders and physical illnesses. Recurrent episodes, high disability rates, and high relapse rates characterize the disease (2). On a global scale, the prevalence of bipolar disorder is relatively high, estimated at approximately 1–2% (3, 4). The most recent World Mental Health Survey, conducted between 2001 and 2022, encompassed 156,331 respondents across 29 countries. The findings revealed a lifetime prevalence of 2.5% for males and 2.3% for females (5). Furthermore, the risk of suicide among patients diagnosed with this disorder is 10 times higher in women than in the general population, while in men it is eight times higher. The findings of this study suggest that bipolar disorder exerts a considerable detrimental effect on the quality of life and survival time of patients (6). According to the most recent classification of the World Health Organization, bipolar disorder has been identified as one of the leading candidates for the global burden of disease in 2030. The disability adjusted life years (DALYs) attributable to this disorder account for 0.5% of the total global disease burden. While neuropsychiatric disorders are projected to account for 15.5% of the total burden of disease by 2030, bipolar disorder is ranked as one of the top five priority disorders for intervention (7, 8). Considering the gravity of the situation, there is an imperative for heightened public awareness regarding the disease, alongside the optimization of diagnostic and treatment systems. Consequently, the dissemination of knowledge about bipolar disorder to the public is of paramount importance.

In recent years, with the rapid development of science and technology, and the emergence of social media (short video platforms) as a major channel for information dissemination, especially in China, the China Internet Network Information Centre (CNNIC) reports that the scale of short video users exceeded 1 billion (1,012 million) for the first time in December 2022, and increased to 1,026 million in June 2023, with a penetration rate of 95.2%. About the intensity of usage, the mean daily usage time of users was 151 min (approximately 2.5 h) in 2023 (9, 10). Short video platforms such as TikTok, Kwai, and Bilibili have disrupted the traditional monopoly on medical information, offering a wider array of sources for the public. This shift has empowered individuals to access medical health information autonomously (11, 12). In contemporary society, the importance of mental health is increasingly recognized, with the public demonstrating growing awareness and understanding of related issues (13–15). Bipolar disorder, as a prevalent mental health issue, is garnering increasing attention. Meanwhile, short video platforms, characterized by their unique fragmented information dissemination methods and high interactivity through features such as comments and retweets, have emerged as one of the primary avenues for individuals to acquire knowledge about bipolar disorder and other mental health topics (16, 17). The persistent stigmatization narratives and ineffective treatment content pushed by short video platforms distort the public’s perception of bipolar disorder, raise the threshold for professional help, and lead to treatment delay and increase the risk of suicide (18, 19). In China, short videos have become the primary source of health information for more than 100 million users and curbing their spread of misinformation is an urgent public health priority. It has been demonstrated that the quality of information accessible to the public regarding mental illnesses, such as schizophrenia and depression, is inconsistent and challenging to regulate (20, 21). For the average user, assessing the scientific validity and reliability of online health information is more difficult, seriously affecting their ability to access quality health knowledge (22–24). Therefore, healthcare professionals need to evaluate the quality of online information related to bipolar disorder to provide appropriate guidance to patients.

According to this study, the quality of online information regarding bipolar disorder on video-based Chinese social media has not been thoroughly investigated. Consequently, we selected several currently popular short video platforms in China, including TikTok, Kwai, Bilibili, WeChat, Xiaohongshu, and Baidu (the video aggregation service of the Baidu search engine), to collect video information related to bipolar disorder. Among these platforms is TikTok, a current representative of emerging short-form video platforms. According to the latest data from Statista (2025), TikTok has approximately 159 million monthly active users, making it the fifth most popular social media platform in the world (25). In addition, TikTok’s website receives approximately 218 million visits per month, with the vast majority of visits (over 65%) coming from mobile devices (26, 27). Kwai is also a highly influential short video platform with a substantial user base, reporting approximately 714 million monthly active users and over 408 million daily active users globally as of the third quarter of 2024 (28). Bilibili is a prominent short-form video platform in China, recognized for its diverse content and interactive features. As of the third quarter of 2024, it has 348 million monthly active users globally, with 83 percent of its user base falling within the 18–24 age group (29). Xiaohongshu is a distinctive short-video and social e-commerce platform that significantly influences the lifestyles of young people through user-generated content and authentic sharing. Furthermore, WeChat and Baidu have demonstrated considerable potential in the short video distribution space in recent years.

Currently, the quality of video information regarding bipolar disorder on most short-form video platforms in China has not been systematically evaluated. This study aims to assess the quality of relevant content available on these platforms and to provide evidence-based recommendations for enhancing health-related short videos.

## Methods

### Ethical considerations

The studies involving human participants were reviewed and approved by the Medical Ethics Committee of Gansu Provincial Hospital. Written informed consent to participate in this study was provided by the participants’ legal guardian/next of kin. The social media data was accessed and analyzed in accordance with the platform’s terms of use and all relevant institutional/national regulations.

### Search strategy and data collection

**Figure 1:**
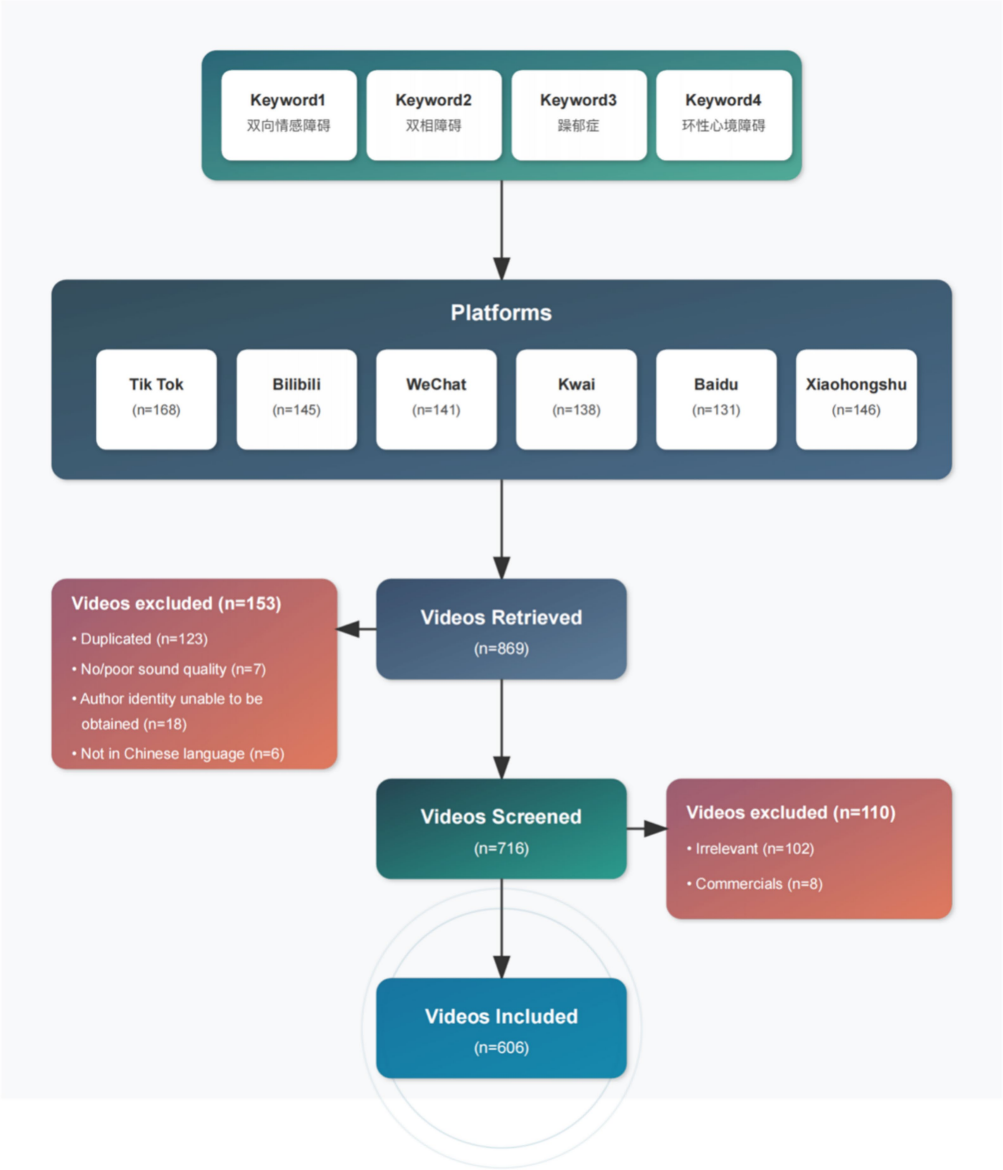
Search strategies for short videos *on* bipolar affective disorder.

In May 2025, we used “双相情感障碍” (bipolar affective disorder), “双相障碍” (bipolar disorder), “躁郁症” (manic depression), and “环性心境障碍” (cyclothymic disorder) as keywords to search on six Chinese social and video platforms and services. We collected the top 100 video results from the dedicated short-video apps of TikTok, Bilibili, Kwai, Xiaohongshu, and WeChat Channels. For Baidu, we conducted searches on the main Baidu search engine (), applied the “Video” tab filter, and collected the top 100 videos from the aggregated results. The default sorting algorithm of each platform was used in the search process, and there was no restriction on the time of video release. We limited our analysis sample to the top 100 videos for two reasons. First, search algorithms on platforms such as TikTok, Kwai, Bilibili, WeChat, Xiaohongshu, and Baidu prioritize content that is highly relevant to the query topic. Videos that rank above 100 are less relevant to bipolar disorder. Secondly, from the perspective of user behavior patterns, most viewers usually only browse the top popular results, rather than all the retrieved content, so the top 100 videos are more representative and more in line with the actual viewing and participation patterns (30, 31). Although content moderation policies may lead to the removal of some videos over time, the cross-sectional approach used in this study enabled the capture and analysis of currently publicly accessible video content at a given point in time. This practice not only ensures the rigor of data collection and repeatability of results, but also truly reflects the information environment that users can access during this period. Exclusion criteria were as follows: (1) repeat video; (2) advertising content; (3) include irrelevant topics; (4) Author information could not be determined (author was anonymous or *did not provide any professional credentials, even self-claimed*); Or (5) poor audio quality. It should be noted that unverified creators claiming to be “doctors” or “psychologists” are still included as independent users, regardless of whether their credentials can be verified, to analyze content that viewers may consider professional. The characteristics of eligible videos were recorded and analyzed, including the number of likes, comments, saves, shares, release time, video duration, video source, the uploader’s address, the number of the uploader’s fans, the video’s presentation form, the video’s content, related diseases, and the medical expertise. All extracted data were entered into Excel (Microsoft Corp) software. The study selection process is summarized in Figure 1.

### Platform selection criteria

**Figure 2:**
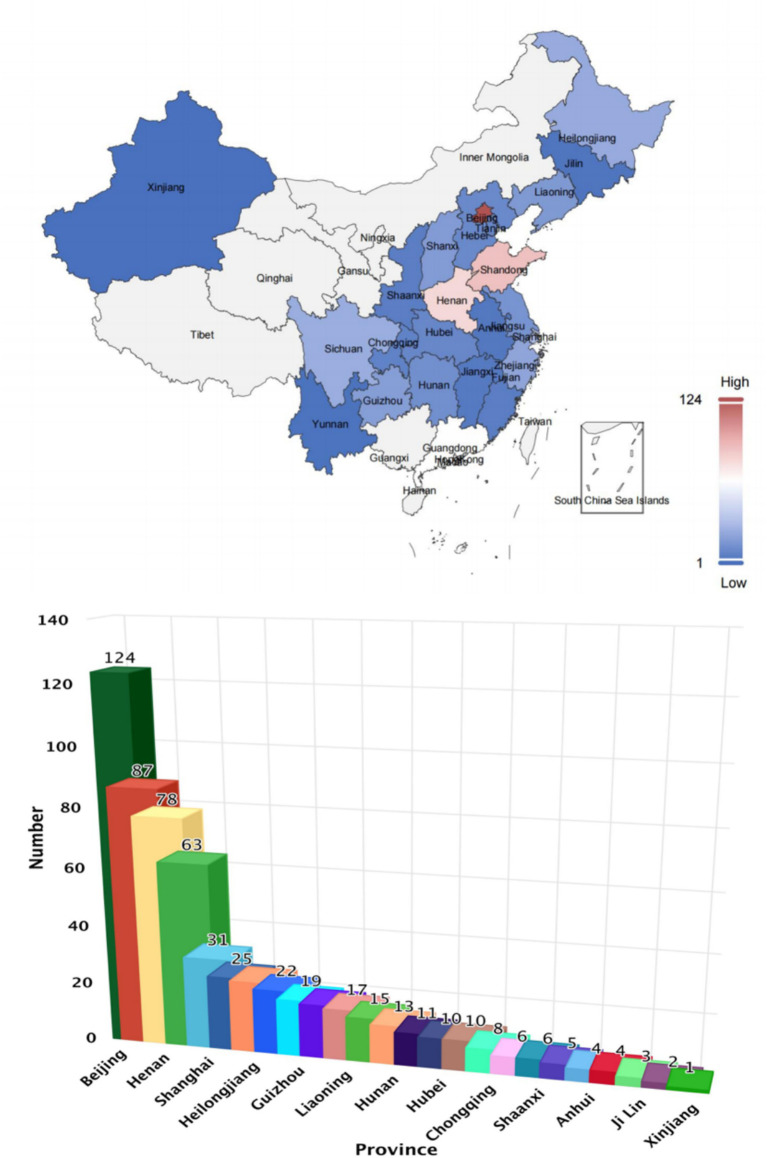
The distribution of video authors in China.

The selection of platforms for this study is based on threefold criteria: (1) User coverage: Platforms ranked among the top 10 in China by monthly activity, including TikTok, Kwai, and Bilibili, were included to ensure the breadth of user coverage. (2) Representativeness of content ecology: This criterion encompasses comprehensive platforms (e.g., WeChat, Baidu), vertical communities (e.g., Xiaohongshu), and generation-specific aggregation platforms (e.g., Bilibili), to comprehensively reflect the characteristics of mental health content dissemination across multiple scenarios. (3) Regional penetration: The selected platforms cover all provincial administrative regions in mainland China and include creators from regions with insufficient medical resources (as shown in Figure 2), thereby avoiding regional biases in the sample.

### Video quality evaluation tools and methods

The JAMA Benchmarking Criteria is a widely used instrument for evaluating the reliability of health-related websites, encompassing four domains: authorship, attribution, disclosure, and currency. GQS is a five-point Likert scale that subjectively rates overall video quality, considering site traffic and usability, with scores ranging from 1 (very poor) to 5 (excellent). The modified DISCERN tool, which is extensively recommended in the literature for assessing the reliability and quality of online resources, comprises five equally weighted items. To further evaluate the videos’ comprehensibility and actionability, we applied the Patient Education Materials Assessment Tool (PEMAT) developed by the Agency for Healthcare Research and Quality (AHRQ). In addition, the reliability and transparency of the online health information were examined using the HONcode. Detailed scoring results for the GQS, JAMA, modified DISCERN, PEMAT, and HONcode assessments are presented in Supplementary Tables 1–5, respectively.

Two psychiatrists with over 10 years of clinical experience independently assessed the standardized process. To minimize potential bias, a strict blinding procedure was implemented. All videos were downloaded and edited using video editing software to remove any on-screen elements that could reveal the creator’s identity (e.g., username, verification badges, profile pictures, hospital logos) or user engagement metrics (e.g., likes, comments, share counts) from the beginning, end, or overlay of the video. Assessors thus evaluated only the core informational content of each video. The scoring strictly followed standardized tools such as GQS, JAMA criteria, modified DISCERN, PEMAT, and HONcode. The evaluation process consisted of the following: first, two evaluators independently scored all videos; second, if there were discrepancies in the scores, a third senior psychiatrist acted as an arbitrator and provided an independent assessment; finally, all three evaluators discussed the discrepant items until a full consensus was reached on the final scores for all assessment items. The *Cohen’s κ* coefficient was used to assess inter-rater agreement for the initial independent ratings, and the result was 0.83 (*p* < 0.001), indicating high inter-rater agreement and excellent reliability.

### Statistical analyses

The data in this study were non-parametric distributions, so descriptive statistics were performed using the median and interquartile range (IQR). The Kruskal-Wallis test was used to evaluate the differences between groups, and then Dunn’s multiple comparison test was used for multiple comparisons. The relationships among quantitative variables were evaluated using the Spearman correlation analysis method. Exploratory regression analyses were performed to assess associations between video variables such as engagement measures, duration, and quality scores. All analyses were performed using R version 4.4.2 (The R Foundation)^1^; Double-tailed *p* < 0.05 is considered statistically significant. These analyses aim to characterize patterns in the data rather than establish predictions or causal relationships, especially given the platform-dependent nature of the engagement metrics.

## Results

### Video characteristics

**Figure 3:**
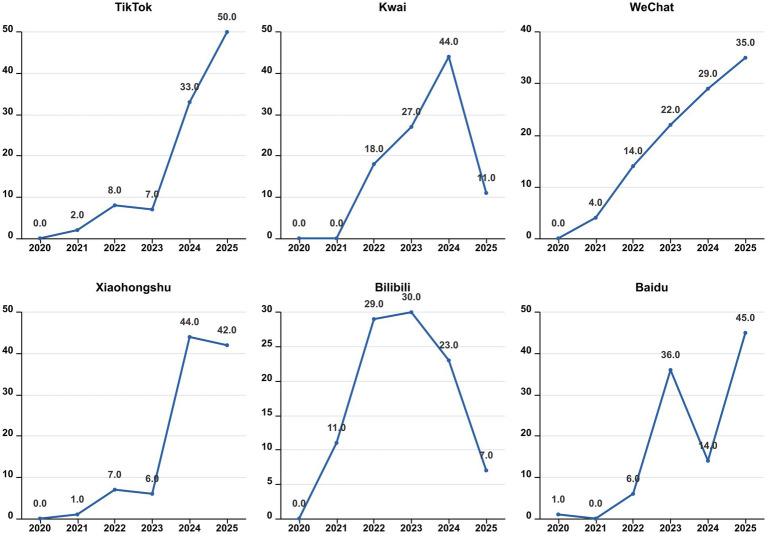
The distribution of video years.

As shown in Table 1, there were statistically significant differences in the number of likes, comments, saves, shares, days since published, video duration, fans, GQS score, JAMA score, Modified DISCERN score, PEMAT Understandability score, Actionability score, and HONCODE score among the six platforms: TikTok, Kwai, WeChat, Xiaohongshu, Bilibili, and Baidu (all *p* < 0.05). Kwai had the highest median numbers of likes and followers, with values of 1,752 and 121,000, respectively. TikTok recorded the highest median numbers of saves and shares, with values of 472 and 475, respectively. WeChat had the highest median number of comments at 495. This difference may be related to the user behavior characteristics of each platform. Kwai users prefer short and relaxed entertainment content and are used to quickly expressing their preferences through likes. With its efficient content dissemination mechanism, TikTok has promoted the saving and sharing of health-related videos related to bipolar disorder. Based on its social network foundation, WeChat is more likely to elicit in-depth comments and exchanges of illness experiences. Bilibili had the longest median video upload time (739 days) and longest video duration (89 min), which is consistent with Bilibili’s positioning as a knowledge-sharing platform, with users preferring longer form and more in-depth content. TikTok’s JAMA score and Modified DISCERN score were both higher than those of videos from the other five platforms, with averages of 2.7 and 3.4, respectively. The highest number of creators originated from Beijing, Henan, and Shanghai, totaling 124, 87, and 78 creators, respectively (Figure 2). Among the videos posted on TikTok, WeChat, and Baidu, the greatest number was uploaded in 2025, with 50, 35, and 45 videos, respectively. In contrast, the highest number of videos on Xiaohongshu and Kwai was posted in 2024, with 44 videos each. For Bilibili, the highest number of videos was released in 2023, totaling 30 (Figure 3).

**Table 1:** General characteristics and scoring of videos related to bipolar affective disorder.

Variable	TikTok	Kwai	WeChat	Xiaohongshu	Bilibili	Baidu	*p*-value
	(*n* = 100)	(*n* = 100)	(*n* = 104)	(*n* = 100)	(*n* = 100)	(*n* = 102)	
Likes (seconds), median (IQR)	1,097 (254–5,084)	1,752 (567–8,549)	251 (62–1,165)	339 (99–893)	262 (70–1,836)	60 (31–151)	<0.001
Comments, median (IQR)	95 (22–734)	274 (64–957)	495 (163–1,572)	25 (5–92)	15 (3–53)	8 (4–16)	<0.001
Saves, median (IQR)	472 (107–1,487)	341 (163–981)	214 (83–758)	130 (35–365)	135 (31–321)	53 (23–148)	0.009
Shares, median (IQR)	475 (78–3,661)	443 (222–1,232)	27 (5–122)	56 (20–225)	32 (12–137)	85 (27–444)	0.001
Days since published, median (IQR)	83 (28–244)	339 (144–698)	281 (78–767)	138 (41–288)	739 (369–894)	230 (53–529)	<0.001
Duration (seconds), median (IQR)	54 (33–73)	45 (21–70)	85 (45–139)	70 (49–129)	89 (50–191)	76 (42–118)	<0.001
Fans, median (IQR)	87,500 (37,500–129,000)	121,000 (58,000–192,750)	64,500 (25,000–232,500)	12,000 (2,088–37,250)	6,992 (1,493–29,000)	34,000 (4,605–97,000)	<0.001
GQS score, mean (SD)	3.4 (0.7)	3.4 (0.9)	3.1 (1.1)	2.8 (0.9)	3.2 (1.1)	2.7 (0.9)	<0.001
JAMA score, mean (SD)	2.7 (0.5)	2.6 (0.9)	2.5 (0.7)	2.4 (0.8)	2.4 (0.7)	2.3 (0.6)	<0.001
Modified DISCERN score, mean (SD)	3.4 (0.8)	3.3 (1.0)	3.0 (0.9)	2.9 (1.0)	3.0 (1.0)	2.8 (0.8)	<0.001
PEMAT Understandability score, mean (SD)	0.7 (0.1)	0.7 (0.1)	0.6 (0.1)	0.6 (0.1)	0.6 (0.1)	0.6 (0.1)	0.002
PEMAT Actionability score, mean (SD)	0.6 (0.1)	0.6 (0.1)	0.6 (0.2)	0.6 (0.1)	0.6 (0.2)	0.6 (0.1)	0.001
HONCODE score, mean (SD)	4.7 (1.6)	4.7 (1.1)	4.5 (1.3)	4.3 (1.3)	4.3 (1.3)	4.1 (1.4)	0.012

JAMA, Journal of the American Medical Association; GQS, Global Quality Scale; PEMAT, Patient Education Materials Assessment Tool; HONCODE, Health on the Net Code.

### Video source and content

As shown in Table 2, there are significant differences among the videos from six different platforms, including their sources, medical specialties, content, knowledge about various diseases, and presentation formats (all *p* < 0.05). Most of the videos on TikTok, Kwai, Xiaohongshu, Bilibili, and Baidu were posted by physicians, accounting for 96, 90, 58, 61, and 79%, respectively. Most of these videos were related to Clinical medicine practitioners, with proportions of 82, 85, 95, 90, 86, and 75%, respectively. In contrast, WeChat videos were predominantly posted by independent users, accounting for 40%. This finding illustrates that professional identity remains key to building trust in the dissemination of health information about bipolar disorder. The relatively high proportion of non-professional content on WeChat may be due to the diversity of its official account ecology, but it also indicates that there is more non-professional information on the platform, which needs to be paid attention to. The video content across TikTok, Kwai, WeChat, Xiaohongshu, Bilibili, and Baidu primarily focused on disease knowledge, with proportions of 90, 78, 82, 83, 81, and 83%, respectively. Regarding disease knowledge, a significant portion of the videos on TikTok, Kwai, WeChat, and Xiaohongshu concentrated on symptoms, accounting for 47, 59, 79, and 60%, respectively. Bilibili primarily addressed symptoms and posttreatment caveats, accounting for 35 and 34%, respectively, while Baidu focused on symptoms and treatment, accounting for 46 and 41%, respectively. This divergence arises from the distinct focus of each platform: content on Bilibili emphasizes “post-treatment precautions,” whereas Baidu concentrates more on the “treatment” protocols themselves. This difference may stem from variations in user intent—Bilibili users are typically seeking more comprehensive knowledge on rehabilitation management, whereas Baidu users are often in the earlier stages of information seeking and thus focus more on diagnosis and treatment options. In terms of video presentation form, TikTok, Kwai, WeChat, Bilibili, and Baidu predominantly featured expert monologs, with proportions of 55, 64, 49, 53, and 70%, respectively. Xiaohongshu utilized expert monologs, visual pictures, and literature, with respective proportions of 42 and 38%. This is highly related to the community culture of Xiaohongshu software.

**Table 2:** Sources and content of videos related to bipolar affective disorder.

Variable	TikTok	Kwai	WeChat	Xiaohongshu	Bilibili	Baidu	*P*-value
	(*n* = 100), n, (%)	(*n* = 100), n, (%)	(*n* = 104), n, (%)	(*n* = 100), n, (%)	(*n* = 100), n, (%)	(*n* = 102), n, (%)	
Video source							<0.001
Physicians	96 (96%)	90 (90%)	24 (23%)	58 (58%)	61 (61%)	81 (79%)	
Hospital	1 (1%)	0 (0%)	14 (13%)	2 (2%)	3 (3%)	2 (2%)	
News agencies	0 (0%)	2 (2%)	24 (23%)	0 (0%)	4 (4%)	5 (5%)	
Independent users	3 (3%)	8 (8%)	42 (40%)	40 (40%)	32 (32%)	14 (14%)	
Different medical specialties							0.002
Clinical medicine practitioner	82 (82%)	85 (85%)	99 (95%)	90 (90%)	86 (86%)	77 (75%)	
TCM practitioner	18 (18%)	15 (15%)	5 (5%)	10 (10%)	14 (14%)	25 (25%)	
Video content							<0.001
Disease knowledge	90 (90%)	78 (78%)	85 (82%)	83 (83%)	81 (81%)	85 (83%)	
Outpatient scenarios	7 (7%)	16 (16%)	2 (2%)	7 (7%)	3 (3%)	7 (7%)	
Personal experience	3 (3%)	6 (6%)	17 (16%)	10 (10%)	16 (16%)	10 (10%)	
Different disease knowledge							<0.001
Treatment	17 (17%)	9 (9%)	11 (11%)	5 (5%)	9 (9%)	42 (41%)	
Symptoms	47 (47%)	59 (59%)	82 (79%)	60 (60%)	35 (35%)	47 (46%)	
Posttreatment caveats	21 (21%)	12 (12%)	1 (1%)	24 (24%)	34 (34%)	3 (3%)	
Prevention	2 (2%)	12 (12%)	2 (2%)	6 (6%)	6 (6%)	6 (6%)	
Definition	13 (13%)	8 (8%)	8 (8%)	5 (5%)	16 (16%)	3 (3%)	
Reexamination	0 (0%)	0 (0%)	0 (0%)	0 (0%)	0 (0%)	1 (1%)	
Video presentation form							<0.001
Expert monolog	55 (55%)	64 (64%)	51 (49%)	42 (42%)	53 (53%)	71 (70%)	
Dialogue	18 (18%)	1 (1%)	9 (9%)	14 (14%)	6 (6%)	13 (13%)	
Video blogs of patients	1 (1%)	4 (4%)	16 (15%)	6 (6%)	11 (11%)	4 (4%)	
Visual pictures and literature	26 (26%)	31 (31%)	28 (27%)	38 (38%)	30 (30%)	14 (14%)	

TCM, traditional Chinese medicine.

### Video quality and reliability assessments

**Figure 4:**
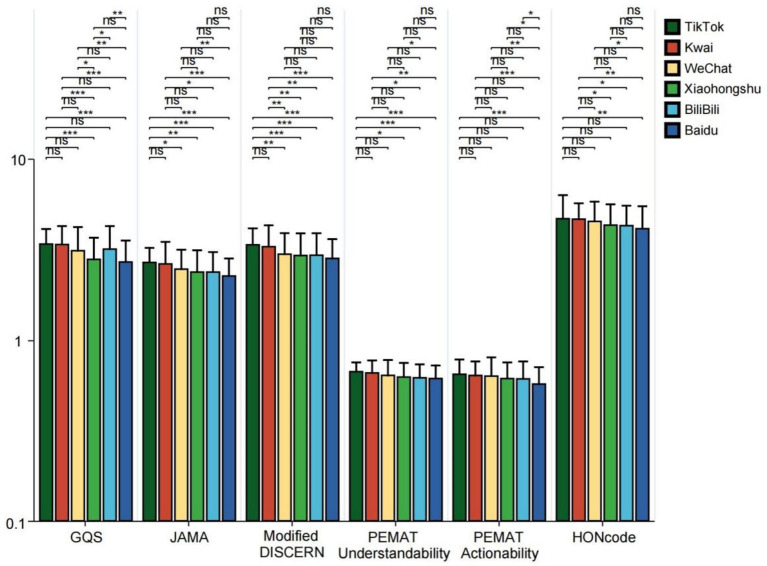
GQS scores, JAMA scores, modified DISCERN scores, PEMAT Understandability score, Actionability score, and HONCODE scores of short videos on bipolar affective disorder on different platforms (TikTok, Kwai, WeChat, Xiaohongshu, Bilibili, and Baidu). *p < 0.05, **p < 0.01, ***p < 0.001. ns: not significant at *p* ≥ 0.05.

In the GQS score, video ratings from TikTok and Kwai were significantly higher than those from Baidu (all *p* < 0.05). In JAMA scores, TikTok’s video ratings surpassed those of WeChat, Xiaohongshu, Bilibili, and Baidu (all *p* < 0.05); the video scores from Baidu and Bilibili were lower than those from Kwai (all *p* < 0.05); furthermore, WeChat’s video scores were higher than those from Baidu (*p* < 0.01). In the Modified DISCERN score, video ratings from TikTok and Kwai were higher than those from WeChat, Xiaohongshu, Bilibili, and Baidu (all *p* < 0.01). Regarding PEMAT Understandability and Actionability scores, Baidu’s video scores were greater than those of TikTok, Kwai, and WeChat (all *p* < 0.05). In terms of HONCODE scores, Baidu’s video ratings were lower than those of TikTok, Kwai, and WeChat (all *p* < 0.05), while Kwai’s video scores exceeded those of Xiaohongshu and Bilibili (all *p* < 0.05) (Figure 4). The higher reliability scores of TikTok and Kwai may be related to the fact that the two platforms have many certified medical professionals to create (Table 2), and the platform mechanism is more conducive to the screening and exposure of high-quality content. However, Baidu’s videos scored the highest in PEMAT Understandability and Actionability, perhaps because their content is more basic and straightforward, aiming to quickly answer users’ questions. In the HONCODE score of information transparency, Baidu was significantly lower than the other platforms, indicating that although it is easy to understand, there are shortcomings in the standardization of citing sources and the disclosure of authors’ qualifications.

### The quality and popularity of videos from different sources, content, and presentation forms

**Figure 5:**
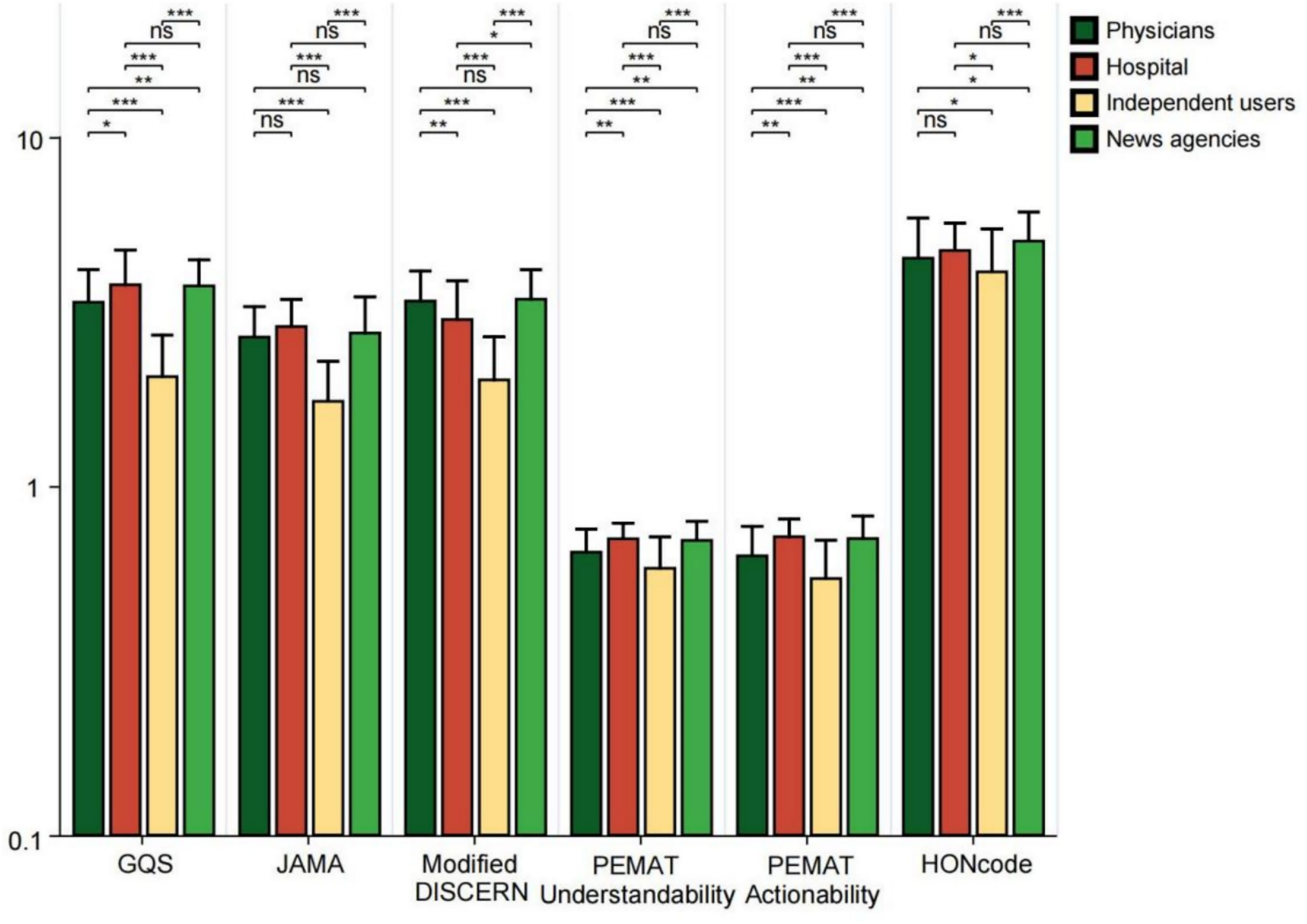
GQS scores, JAMA scores, modified DISCERN scores, PEMAT Understandability score, Actionability score, and HONCODE scores from different sources of videos related to bipolar affective disorder. *p < 0.05, **p < 0.01, ***p < 0.001. ns: not significant at *p* ≥ 0.05.

**Figure 6:**
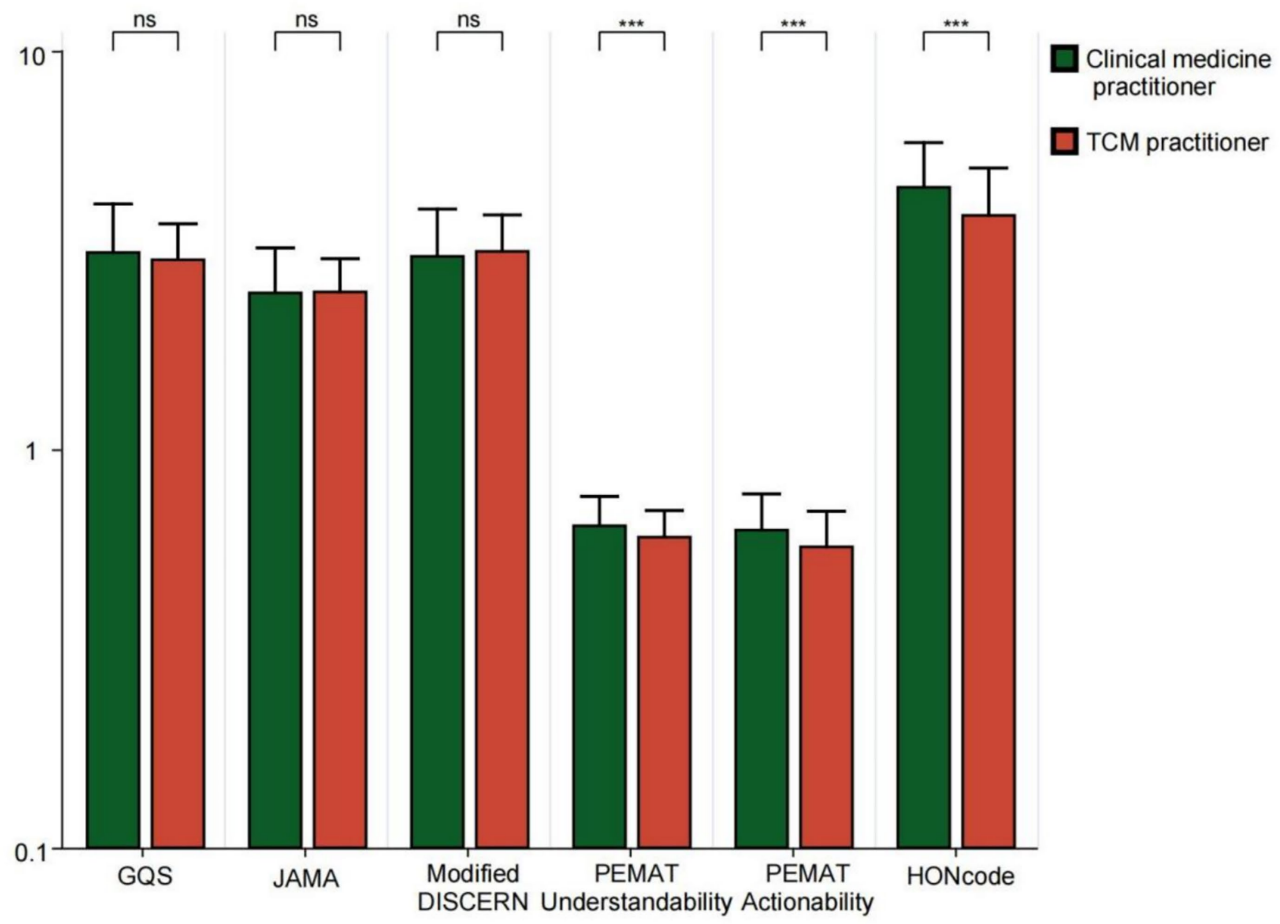
GQS scores, JAMA scores, modified DISCERN scores, PEMAT Understandability score, Actionability score, and HONCODE scores for bipolar affective disorder*-*related videos from different medical specialties. *p < 0.05, **p < 0.01, ***p < 0.001. ns: not significant at *p* ≥ 0.05.

**Figure 7:**
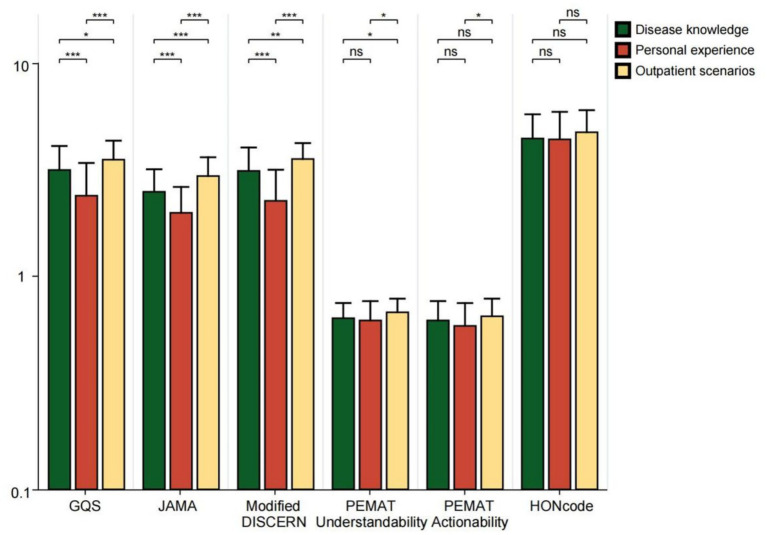
GQS scores, JAMA scores, modified DISCERN scores, PEMAT Understandability score, Actionability score, and HONCODE scores for bipolar affective disorder-related videos with different content. **p < 0.01, ***p < 0.001. *ns: not significant at *p* ≥ 0.05.

Videos posted by physicians, hospitals, and news agencies received significantly higher ratings than those from independent users across GQS, JAMA, Modified DISCERN, PEMAT (Understandability and Actionability), and HONCODE assessments (all *p* < 0.05). News agencies scored higher than physicians in GQS, PEMAT (Understandability and Actionability), and HONCODE ratings (all *p* < 0.05) (Figure 5). This shows that the source of the video is a key factor in predicting its quality. Content released by professional organizations is more rigorously reviewed, more scientific, and of higher quality. News organizations are better at colloquia and good production and thus perform better in user experience (GQS) and intelligibility (PEMAT). Among the videos uploaded by physicians, the PEMAT Understandability score, Actionability score, and HONCODE score for videos posted by clinical medicine practitioners were significantly higher than those posted by TCM practitioners (all *p* < 0.001) (Figure 6). For bipolar disorder, the treatment of modern Western medicine and popular science content is more international and standardized, often with higher accuracy and clarity. TCM often scores low in standardized assessments due to its traditional expression and integration of multiple concepts. Additionally, the GQS score, JAMA score, Modified DISCERN score, and PEMAT Understandability score for different video content were significantly higher for outpatient scenario videos compared to those that focused on personal experiences and disease knowledge (all *p* < 0.05) (Figure 7). This may be since the outpatient scene significantly improves the professional credibility of the content and the sense of situational substitution by restoring the real diagnosis and treatment process, which is an efficient form of knowledge transmission. In contrast, disease knowledge explanation was difficult to go in-depth due to time limitations, and personal experience sharing was subjective and prone to containing atypical information, so the quality score was generally low.

**Figure 8:**
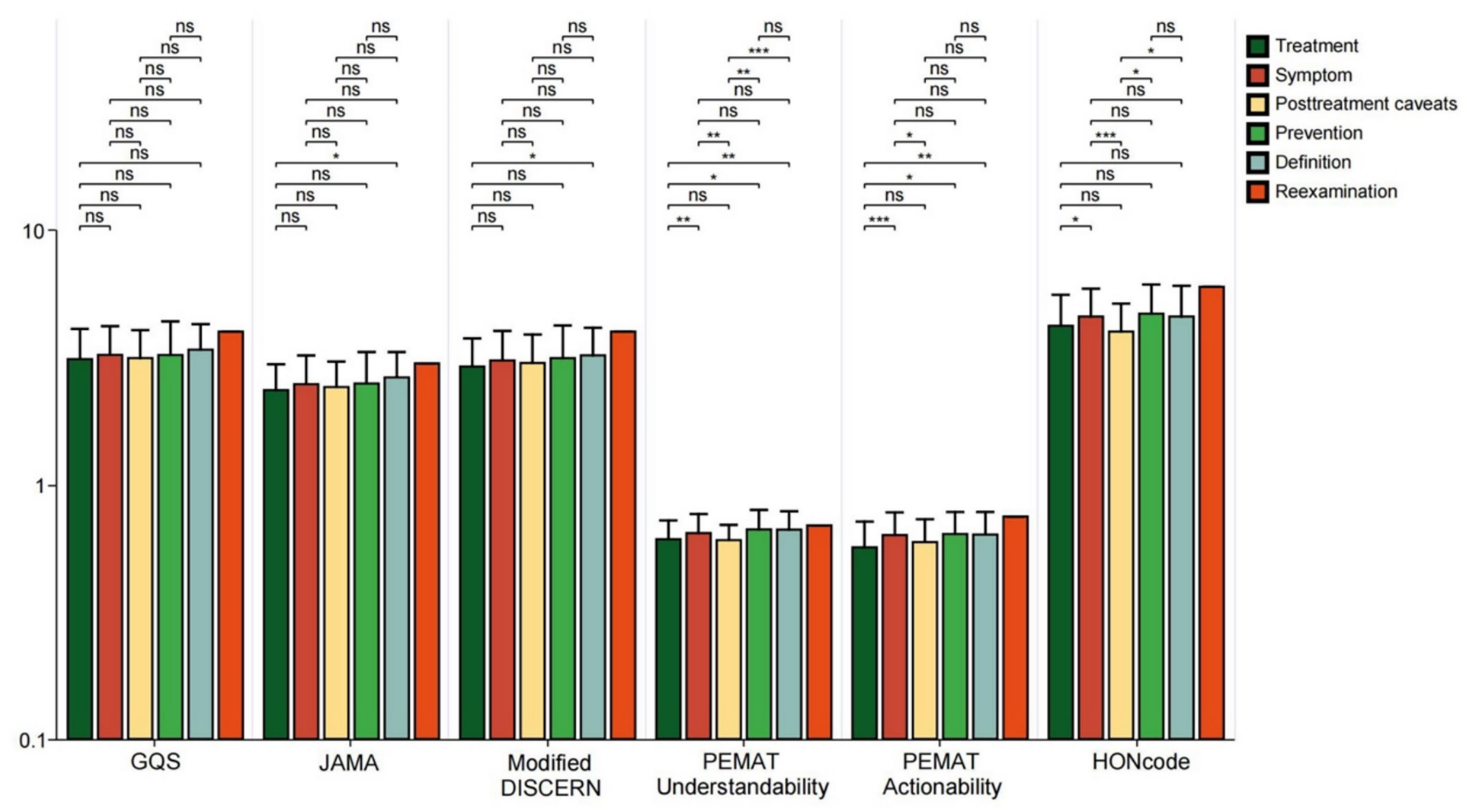
GQS scores, JAMA scores, modified DISCERN scores, PEMAT Understandability score, Actionability score, and HONCODE scores for bipolar affective disorder-related videos for different disease knowledge. *p < 0.05. *ns: not significant at *p* ≥ 0.05.

**Figure 9:**
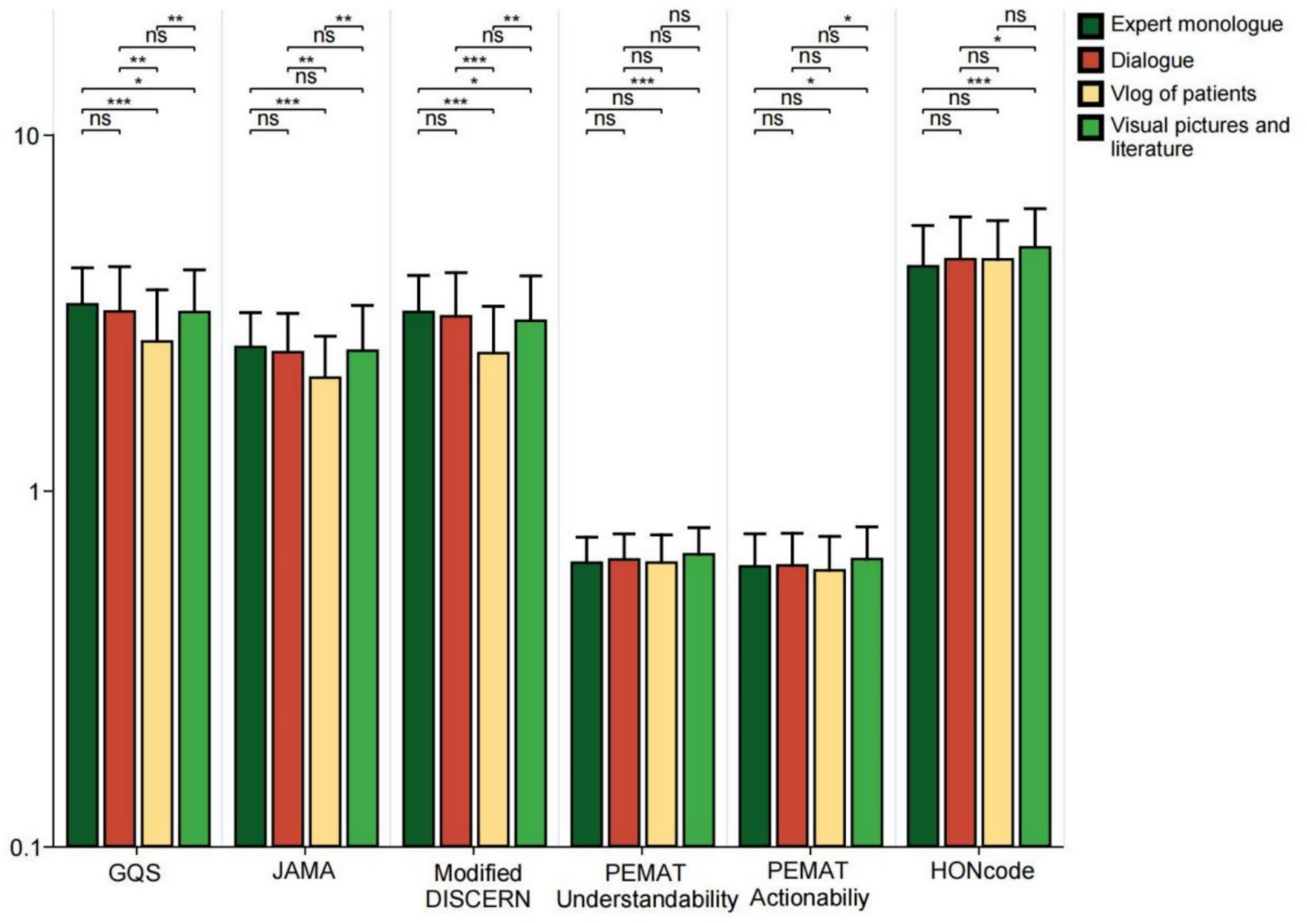
GQS scores, JAMA scores, modified DISCERN scores, PEMAT Understandability score, Actionability score, and HONCODE scores for bipolar affective disorder*-*related videos for different presentation forms. *p < 0.05, **p < 0.01, ***p < 0.001. *ns: not significant at *p* ≥ 0.05.

In videos focused on disease knowledge, the JAMA scores and Modified DISCERN scores for treatment-related videos were lower than those for definition-related videos (all *p* < 0.05). The HONCODE scores showed that the treatment videos were rated lower than the symptom videos (*p* < 0.05) and the videos on post-treatment precautions were rated lower than the symptom, prevention, and definition videos (all *p* < 0.05) (Figure 8). Among videos featuring different presentation forms, GQS scores, JAMA scores, and Modified DISCERN scores were significantly lower for patient video blogs compared to expert monologs, dialogues, Visual pictures, and literature (all *p* < 0.05). Regarding the PEMAT Understandability score, expert monolog videos received lower ratings than those of Visual pictures and literature (*p* < 0.05). In terms of actionability scores, both expert monolog videos and patient blogs were rated lower than Visual pictures and literature (all *p* < 0.05). Additionally, HONCODE scores for expert monolog and dialogue videos were lower than those for Visual pictures and literature (all *p* < 0.05) (Figure 9). This reveals a key paradox: high-quality content does not necessarily lead to high engagement. Patient video blog has a strong story and is easy to cause emotional interaction, but its quality score is the lowest due to subjectivity and non-professionalism. The form of “Visual pictures and literature “makes the content clear and easy to understand with high quality through intuitive charts and literature citations. At the same time, because of its dense information and strong practicality, users are more willing to collect and share, to achieve an effective balance between quality and dissemination.

As shown in Table 3, the popularity of videos, measured by likes, comments, saves, and shares, was compared across different sources, medical specialties, content types, disease knowledge, and presentation form. The results indicated that among various sources, videos produced by independent users received more likes and saves, videos from news agencies garnered more comments, and physicians ‘videos were shared more frequently. Additionally, videos related to clinical medicine practitioners showed a higher number of likes, comments, and saves (all *p* < 0.01). In terms of video content, personal experience videos were more likely to be liked (*p* = 0.007). Regarding disease knowledge, videos covering preventive measures, symptoms, and definitions outperformed others in terms of likes, comments, and saves (all *p* < 0.05). In terms of video presentation, patient video blogs performed better in terms of likes and comments, while videos featuring Visual pictures and literature were more likely to be saved and shared (all *p* < 0.05).

**Table 3:** The popularity of videos from different sources with different content and presentation forms.

Variables	Likes	Comments	Saves	Shares
Video source				
Physicians, median (IQR)	390 (93–1,665)	38 (9–234)	161 (46–591)	166 (33–644)
Hospital, median (IQR)	78 (34–231)	100 (16–172)	101 (62–175)	24 (3–60)
News agencies, median (IQR)	223 (46–3,263)	197 (67–1,061)	179 (41–939)	30 (5–152)
Independent users, median (IQR)	586 (124–2,853)	94 (10–628)	234 (78–850)	62 (23–290)
*P*-value	<0.001	<0.001	0.047	<0.001
Different medical specialties				
Clinical medicine practitioner, median (IQR)	482 (98–2,706)	67 (12–450)	200 (60–791)	119 (26–612)
TCM practitioner, median (IQR)	151 (46–433)	15 (5–49)	70 (30–200)	51 (16–261)
*P*-value	<0.001	<0.001	<0.001	0.007
Video content				
Disease knowledge, median (IQR)	308 (74–1,520)	47 (10–275)	158 (51–647)	111 (23–493)
Outpatient scenarios, median (IQR)	540 (195–3,779)	46 (16–658)	222 (57–565)	147 (36–816)
Personal experience, median (IQR)	1,025 (176–3,962)	112 (9–796)	254 (88–932)	55 (28–586)
*P*-value	0.007	0.167	0.187	0.425
Different disease knowledge				
Treatment, median (IQR)	172 (44–639)	19(5–99)	97(35–297)	56(20–487)
Symptoms, median (IQR)	492 (104–2,858)	84 (15–574)	210(69–766)	128(26–603)
Precautions after treatment, median (IQR)	275(90–861)	20(5–80)	116(40–246)	59(26–228)
Prevention, median (IQR)	796(174–4,200)	49(9–462)	206(62–891)	126(32–917)
Definition, median (IQR)	732(111–4,785)	72(12–420)	250(58–1,082)	252(24–811)
Reexamination, median (IQR)	211(211–211)	43 (43–43)	51(51–51)	176(176–176)
*P*-value	<0.001	<0.001	<0.001	0.065
Video presentation form				
Expert monolog, median (IQR)	238 (64–1,062)	36 (8–196)	126 (43–368)	96 (23–438)
Dialogue, median (IQR)	223 (89–810)	25 (10–103)	136 (39–502)	67 (22–261)
Video blogs of patients, median (IQR)	1,008 (271–3,430)	131 (16–739)	252 (107–932)	54 (26–319)
Visual pictures and literature, median (IQR)	903 (162–5,641)	131 (20–694)	308 (92–1,153)	208 (28–850)
*P*-value	<0.001	<0.001	<0.001	0.011

TCM, traditional Chinese medicine.

### Correlation analysis

**Figure 10:**
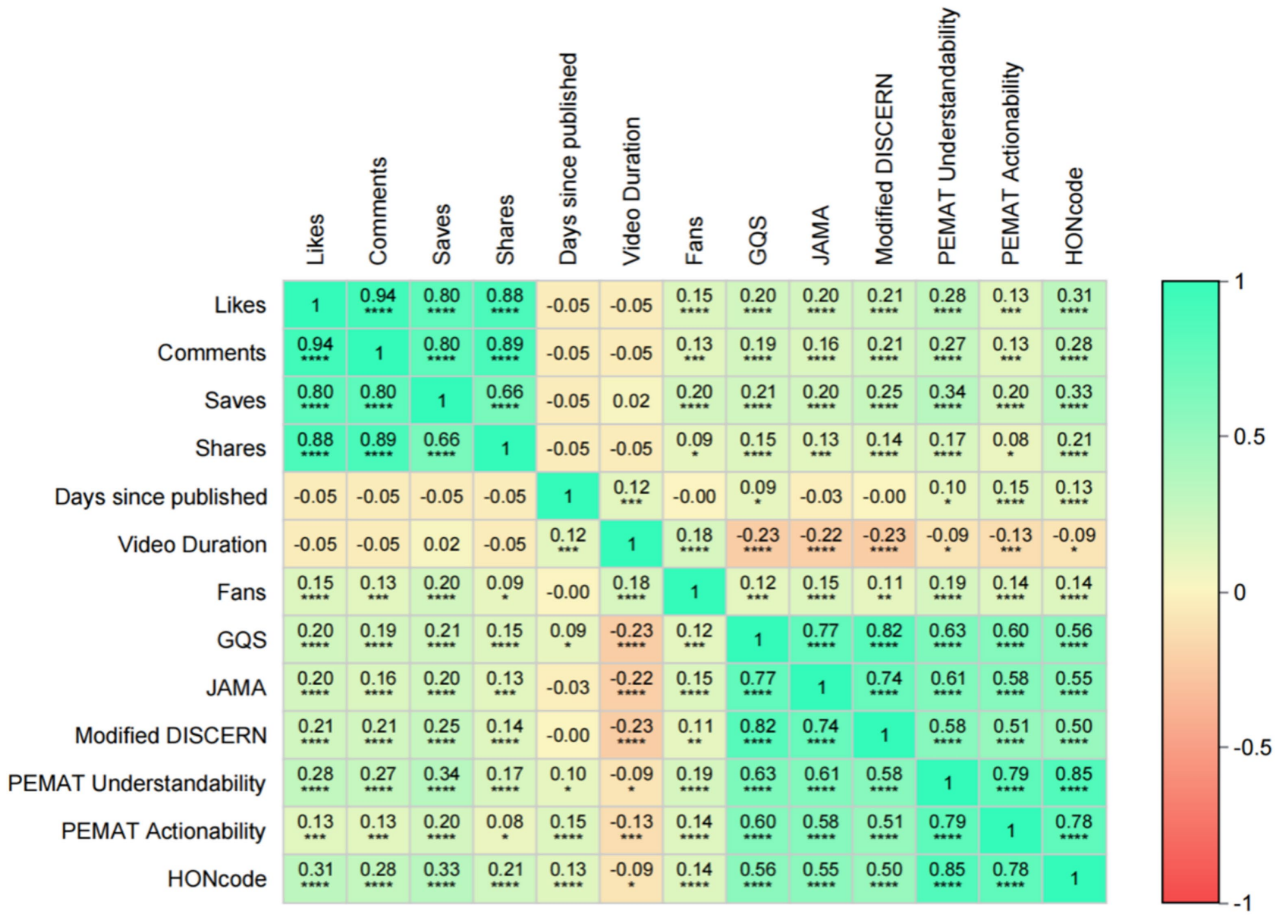
Spearman correlation analyses between video variables and between video variables and GQS scores, JAMA scores, modified DISCERN scores, PEMAT Understandability score, Actionability score, and HONCODE scores. *p < 0.05, **p < 0.01, ***p < 0.001, ****p < 0.0001.

The results of Spearman correlation analysis showed that there were statistically significant positive correlations between the number of likes, comments, favorites, and shares (all *p* < 0.001; Spearman’s rho coefficient ranged from 0.66 to 0.94). There were also statistically significant positive associations between these interaction indicators and the assessment scores (GQS, JAMA, modified DISCERN, PEMAT intelligibility, and operability scores) (all *p* < 0.05). It is important to note that this observed association does not imply causation; videos with higher interaction metrics may not necessarily have higher quality, and vice versa. However, Effect sizes were generally weak to moderate (Spearman’s rho coefficient ranged from 0.09 to 0.34). In contrast, video duration showed consistent, statistically significant, and weak negative associations with all assessment scores (*p* < 0.05 for all; rho coefficients range, approximately − 0.09 to − 0.23), which indicates that longer videos tend to receive slightly lower quality scores. The different scoring systems themselves had strong positive correlations (*p* < 0.001 for all; rho coefficients ranged from 0.50 to 0.85), which indicates their high agreement in assessing video quality (Figure 10). The negative correlation between video length and quality is a noteworthy finding. One possible explanation is that excessively long content tends to be difficult to keep compact on short video platforms, which may lead to lengthy and distracting information and reduced viewer attention, thus causing lower ratings. However, other explanations still need to be considered, such as the fact that popular creators may tend to produce shorter content—regardless of its actual quality—and that these types of videos are generally more likely to garner higher engagement, which could also be a contributing factor to the phenomenon. However, the association may also be influenced by platform characteristics and assessment tools. For example, Bilibili, which is dominated by long videos, has a significantly higher average duration than TikTok and Kwai, but lower quality scores, such as JAMA and Modified DISCERN (Table 1). This negative correlation may be more a reflection of systematic differences in content between platforms rather than a direct quality decline due to duration. In addition, the assessment tool used was originally designed for text and was not sensitive enough to the narrative and educational value of long videos to objectively evaluate content that requires in-depth interpretation.

### Regression analysis of video variables and video quality

Exploratory analyses showed that all video quality scores were significantly correlated with interaction behaviors (liking, commenting, saving, and sharing; Supplementary Figure 1) and video duration (Supplementary Figure 2) (all *p* < 0.05); however, due to the platform-dependent nature of the interaction metrics, these associations were limited to descriptive correlations and are not suitable for outward generalization.

## Discussion

### Principal findings

This study analyzed the characteristics and quality of videos related to bipolar disorder across multiple platforms. Significant differences were observed in both audience engagement metrics and video attributes among the platforms. Specifically, in terms of engagement, Kwai had the highest median number of likes and fans, while TikTok excelled in terms of saves and shares. WeChat received a significantly greater number of comments compared to other platforms. In contrast, Bilibili exhibited the longest upload duration and the longest video length. Separately, when assessing informational quality, TikTok recorded higher scores across all five metrics compared to other platforms. These differences in engagement reflect the behavioral preferences and content dissemination characteristics of users on various platforms. The differences in quality scores reflect varying levels of adherence to scientific and editorial standards across platforms. Consistent with previous research (30), we found that low-quality mental health content contributed to misperceptions. As shown in Figure 5, videos posted by nonexpert creators generally scored lower on scientific accuracy, most likely perpetuating harmful stereotypes. The results again echo WHO’s call for digital platforms for mental health governance, for which platform-level interventions are urgently needed. Therefore, having a medical background is crucial, and it is even more essential to disseminate medical knowledge in a scientifically accurate manner. Additionally, the impact of video content created by professionals should be carefully evaluated (32). Secondly, the public must become more aware of the importance of utilizing reliable sources for obtaining health information to ensure that their understanding of diseases is not compromised by the varying quality of available content (33).

We contrasted the findings of this study with previous studies on video quality assessment in mental health, such as depression and schizophrenia (20, 21). Consistent with previous studies, the quality of health information in short video platforms is generally heterogeneous, and a similar trend was observed in BD related content in this study. However, we also identified some challenges unique to the disease: low public awareness and strong stigma (34), prompting authors to focus more on symptom interpretation and misunderstanding clarification; The bipolarity of its clinical manifestations (mania and depression) easily leads to sensationalized narratives, which aggravates the “quality and prevalence paradox.” The complexity of diseases also increases the difficulty of short video popularization. This study not only verified the general lack of quality of short video health information but also provided new specific insights for bipolar disorder.

This study found three main problems with how short videos share information about bipolar disorder. First, longer videos tend to receive lower quality ratings. Because bipolar disorder is complex, it is hard to explain fully in a short video. To keep videos brief, creators often simplify too much or leave out important details. This reduces the completeness and accuracy of the information, which lowers overall quality. Second, high-quality videos do not always get more engagement. Videos made by health professionals are more reliable and scientific, but personal vlogs by patients get more likes and comments. This “quality–popularity paradox” might occur because emotional and story-based content is shared more widely. Furthermore, platform algorithms are known to promote videos with more interactions, which could potentially amplify the reach of less scientific but more attractive content. Therefore, although the scientific quality of personal vlogs is lower, their storytelling expression is more likely to elicit high interaction. The feedback loop between algorithms and users may allow emotional content to get over-disseminated regardless of information accuracy. Interaction data should be viewed as an indicator of prevalence, not as a basis for scientific quality. High interaction does not equal high scientific validity, which requires the attention of users, creators, and platforms. Third, most video creators live in big cities like Beijing and Shanghai, where medical resources are plentiful. This means users in areas with fewer medical resources may have less access to good-quality information. Instead of reducing this inequality, short video platforms may be making regional health knowledge gaps worse.

### Recommendations based on our results

Based on the findings of this study, we propose the following suggestions to improve the quality and dissemination of short videos related to bipolar disorder. Short video platforms should improve content recommendation algorithms by including quality metrics, not just interaction metrics, to increase the visibility of trusted information. They should also validate the credentials of medical creators through accreditation systems and provide evidence-based content guidelines that encourage the citation of authoritative sources. In addition, platforms should avoid restricting the flow of medically sensitive terms during the audit process, aiming to improve the efficiency of the dissemination of professional information, while ensuring that the content remains scientific and reliable. Healthcare professionals are advised to use clinical scenarios or visual AIDS such as animations and diagrams when producing videos to increase clarity and engagement, and to break down complex topics into short, focused segments. Viewers should critically evaluate sources, choose content from certified providers, and be wary of videos that rely heavily on personal stories or exaggerations. Finally, health organizations and policymakers should support the establishment of quality certification systems for BD related content and provide more training and resources for creators in underserved areas to reduce information inequity.

### Strengths and limitations

The strength of this study lies in its comprehensive analysis of the quality of bipolar BD-related videos across six short-form video platforms, which examines the relationship between video quality and prevalence. However, several limitations should be noted. First, the analysis was restricted to six Chinese short-video platforms, limiting the generalizability of the findings to other social media platforms. In addition, because this study was conducted in China’s specific sociocultural and digital ecosystem, the findings may not be directly generalizable to other countries with different cultural attitudes toward mental health, healthcare systems, and platform governance policies. Second, approximately 100 relevant videos were selected from each platform, which may not fully represent the entire population of BD-related content available on such platforms. Third, the use of a cross-sectional design, as opposed to a cohort design, precludes the establishment of causal relationships. Fourth, the cross-sectional design of this study suggests that the relationships found between video quality and engagement measures, such as likes and shares, are only correlational and cannot establish causality. Longitudinal or experimental studies are needed to test the causal effect of video quality on engagement. Finally, the assessment tools used in this study (e.g., GQS, JAMA Benchmarks, etc.) were originally designed for text-based health messages (e.g., websites) and did not cover key features of short videos, such as audio-visual elements, fragmented representations, and duration constraints. This mismatch between tool and medium may introduce assessment bias. This issue is particularly acute in this field, especially in the Chinese context, where there is still a lack of specifically validated video assessment tools. Although we adapted existing tools to create a standardized assessment framework, which has been a common practice in the past, the development of validated native tools designed specifically for health-based short videos remains key to improving the measurement validity of future studies.

## Conclusion

This study reveals that although clinical practitioners are the primary creators of bipolar disorder-related content on Chinese short-video platforms, often using expert-led monologs, the overall scientific depth and production quality remain inadequate. Significant inter-platform variability exists, with TikTok and Kwai showing higher reliability scores, while Baidu and WeChat offered more understandable but less transparently sourced content. A clear “quality–popularity paradox” was observed, whereby emotionally compelling yet scientifically weak videos achieved wider engagement. Furthermore, geographic disparities in content creation may worsen existing inequities in access to reliable mental health information. These findings underscore the need for improved content supervision, creator training, and algorithm refinement to promote accurate, accessible information on bipolar disorder.

## Data Availability

The raw data supporting the conclusions of this article will be made available by the authors, without undue reservation.

## Footnotes

^1^ http://www.R-project.org

## References

[1] First MB. Diagnostic and statistical manual of mental disorders, 5th edition, and clinical utility. J Nerv Ment Dis. (2013) 201:727–9. doi: 10.1097/NMD.0b013e3182a2168a, PMID: 23995026

[2] Luo J Liang M Yi P Li X. The neuropsychological mechanisms of treatment of bipolar disorder and borderline personality disorder: activation likelihood estimation meta-analysis of brain imaging research. J Clin Psychiatry. (2023) 84:22r14463. doi: 10.4088/JCP.22r14463., PMID: 36988478

[3] Merikangas KR Jin R He JP Kessler RC Lee S Sampson NA. Prevalence and correlates of bipolar spectrum disorder in the world mental health survey initiative. Arch Gen Psychiatry. (2011) 68:241–51. doi: 10.1001/archgenpsychiatry.2011.12, PMID: 21383262 PMC3486639

[4] Humpston CS Bebbington P Marwaha S. Bipolar disorder: prevalence, help-seeking and use of mental health care in England. Findings from the 2014 adult psychiatric morbidity survey. J Affect Disord. (2021) 282:426–33. doi: 10.1016/j.jad.2020.12.151., PMID: 33422818

[5] McGrath JJ Al-Hamzawi A Alonso J Altwaijri Y Andrade LH Bromet EJ. Age of onset and cumulative risk of mental disorders: a cross-national analysis of population surveys from 29 countries. Lancet Psychiatry. (2023) 10:668–81. doi: 10.1016/s2215-0366(23)00193-1, PMID: 37531964 PMC10529120

[6] Crump C Sundquist K Winkleby MA Sundquist J. Comorbidities and mortality in bipolar disorder: a Swedish national cohort study. JAMA Psychiatr. (2013) 70:931–9. doi: 10.1001/jamapsychiatry.2013.1394, PMID: 23863861

[7] Whiteford HA Degenhardt L Rehm J Baxter AJ Ferrari AJ Erskine HE. Global burden of disease attributable to mental and substance use disorders: findings from the global burden of disease study 2010. Lancet. (2013) 382:1575–86. doi: 10.1016/s0140-6736(13)61611-6, PMID: 23993280

[8] World Health Organization. Mental health action plan 2013-2030. Geneva, Switzerland: World Health Organization (2021).

[9] China Internet Network Information Center (CNNIC). The 51st statistical report on internet development in China. Beijing, China: CNNIC (2023).

[10] National Radio and Television Administration (NRTA). Research report on the development of China’s online audiovisual industry. Beijing, China: NRTA (2024).

[11] Ventola CL. Social media and health care professionals: benefits, risks, and best practices. P T. (2014) 39:491–520. 25083128 PMC4103576

[12] Moorhead SA Hazlett DE Harrison L Carroll JK Irwin A Hoving C. A new dimension of health care: systematic review of the uses, benefits, and limitations of social media for health communication. J Med Internet Res. (2013) 15:e85. doi: 10.2196/jmir.1933, PMID: 23615206 PMC3636326

[13] Patel V Saxena S Lund C Thornicroft G Baingana F Bolton P. The lancet commission on global mental health and sustainable development. Lancet. (2018) 392:1553–98. doi: 10.1016/s0140-6736(18)31612-x, PMID: 30314863

[14] Corrigan PW Druss BG Perlick DA. The impact of mental illness stigma on seeking and participating in mental health care. Psychol Sci Public Interest. (2014) 15:37–70. doi: 10.1177/1529100614531398, PMID: 26171956

[15] Vigo D Thornicroft G Atun R. Estimating the true global burden of mental illness. Lancet Psychiatr. (2016) 3:171–8. doi: 10.1016/s2215-0366(15)00505-2, PMID: 26851330

[16] Naslund JA Aschbrenner KA Marsch LA Bartels SJ. The future of mental health care: peer-to-peer support and social media. Epidemiol Psychiatr Sci. (2016) 25:113–22. doi: 10.1017/s2045796015001067, PMID: 26744309 PMC4830464

[17] Engel E Gell S Heiss R Karsay K. Social media influencers and adolescents' health: a scoping review of the research field. Soc Sci Med. (2024) 340:116387. doi: 10.1016/j.socscimed.2023.116387, PMID: 38039770

[18] Mayor E Bietti LM. A social media study of portrayals of bipolar disorders on YouTube: content and thematic analyses. J Med Internet Res. (2025) 27:e67129. doi: 10.2196/67129, PMID: 40279634 PMC12064968

[19] Srivastava K Chaudhury S Bhat PS Mujawar S. Media and mental health. Ind Psychiatry J. (2018) 27:1–5. doi: 10.4103/ipj.ipj_73_18, PMID: 30416284 PMC6198586

[20] Zhang W Wang M Shu H Zhou C Zhang C Hu C. Evaluation of the content and quality of schizophrenia on TikTok: a cross-sectional study. Sci Rep. (2024) 14:26448. doi: 10.1038/s41598-024-75372-7, PMID: 39488554 PMC11531578

[21] Chen Y Yin J Ding Y Wang C Zhu J Niu L. Evaluation of the quality of depression-related information on Chinese websites and video platforms: a cross-sectional comparative analysis. Front Psych. (2024) 15:1408384. doi: 10.3389/fpsyt.2024.1408384, PMID: 39726915 PMC11669601

[22] Diviani N van den Putte B Giani S van Weert JC. Low health literacy and evaluation of online health information: a systematic review of the literature. J Med Internet Res. (2015) 17:e112. doi: 10.2196/jmir.4018, PMID: 25953147 PMC4468598

[23] Kata A. A postmodern Pandora's box: anti-vaccination misinformation on the internet. Vaccine. (2010) 28:1709–16. doi: 10.1016/j.vaccine.2009.12.022, PMID: 20045099

[24] Zhao Y Zhang J. Consumer health information seeking in social media: a literature review. Health Inf Libr J. (2017) 34:268–83. doi: 10.1111/hir.12192, PMID: 29045011

[25] Statista. Number of monthly active TikTok users worldwide from January 2018 to March 2025. Statista GmbH (2025). Available online at: https://www.statista.com/statistics/1234567/tiktok-global-mau.

[26] BusinessofApps. TikTok statistics and facts. BusinessofApps Ltd; (2025). Available onlinen at: https://www.businessofapps.com/data/tiktok-statistics/.

[27] SimilarWeb. TikTok analytics: monthly visits, user demographics, and more. SimilarWeb Ltd. (2025). Available online at: https://www.similarweb.com/website/tiktok.com/.

[28] Technology K. (2024) Third quarter financial report. Kuaishou Inc. Available online at: https://ir.kuaishou.com/news-releases/news-release-details/kuaishou-technology-announces-third-quarter-2024-unaudited/.

[29] Inc B. Third quarter financial report. Bilibili (2024). Available online at: https://www.globenewswire.com/news-release/2024/11/14/2980919/0/en/Bilibili-Inc-Announces-Third-Quarter-2024-Financial-Results.html.

[30] Li M Liang RB Zhou Y Wang K Yang R Li T. Assessing the quality of myopia prevention videos on Chinese short video platforms: a cross-sectional content analysis by source. BMJ Open. (2025) 15:e102818. doi: 10.1136/bmjopen-2025-102818, PMID: 40840988 PMC12374652

[31] Zhu W He B Wang X Du Y Young K Jiang S. Information quality of videos related to esophageal cancer on tiktok, kwai, and bilibili: a cross-sectional study. BMC Public Health. (2025) 25:2245. doi: 10.1186/s12889-025-23475-9, PMID: 40604838 PMC12220660

[32] Pretorius C McCashin D Coyle D. Mental health professionals as influencers on TikTok and Instagram: what role do they play in mental health literacy and help-seeking? Internet Interv. (2022) 30:100591. doi: 10.1016/j.invent.2022.100591, PMID: 36458161 PMC9706523

[33] Sumayyia MD Al-Madaney MM Almousawi FH. Health information on social media. Perceptions, attitudes, and practices of patients and their companions. Saudi Med J. (2019) 40:1294–8. doi: 10.15537/smj.2019.12.24682, PMID: 31828284 PMC6969626

[34] Latifian M Abdi K Raheb G Islam SMS Alikhani R. Stigma in people living with bipolar disorder and their families: a systematic review. Int J Bipolar Disord. (2023) 11:9. doi: 10.1186/s40345-023-00290-y, PMID: 36805368 PMC9941403

