# Supplementary material for: Videos in short video sharing platforms as a source of information on bipolar disorder: a cross-sectional content analysis study
Source: Front Public Health. 2025 Oct 28;13:1627885. doi: 10.3389/fpubh.2025.1627885 (PMC12602428; doi:10.3389/fpubh.2025.1627885)
Supplement: Supplementary file 1 [file Data_Sheet_1.zip › supplementary material/Supplementary figure 2.docx]

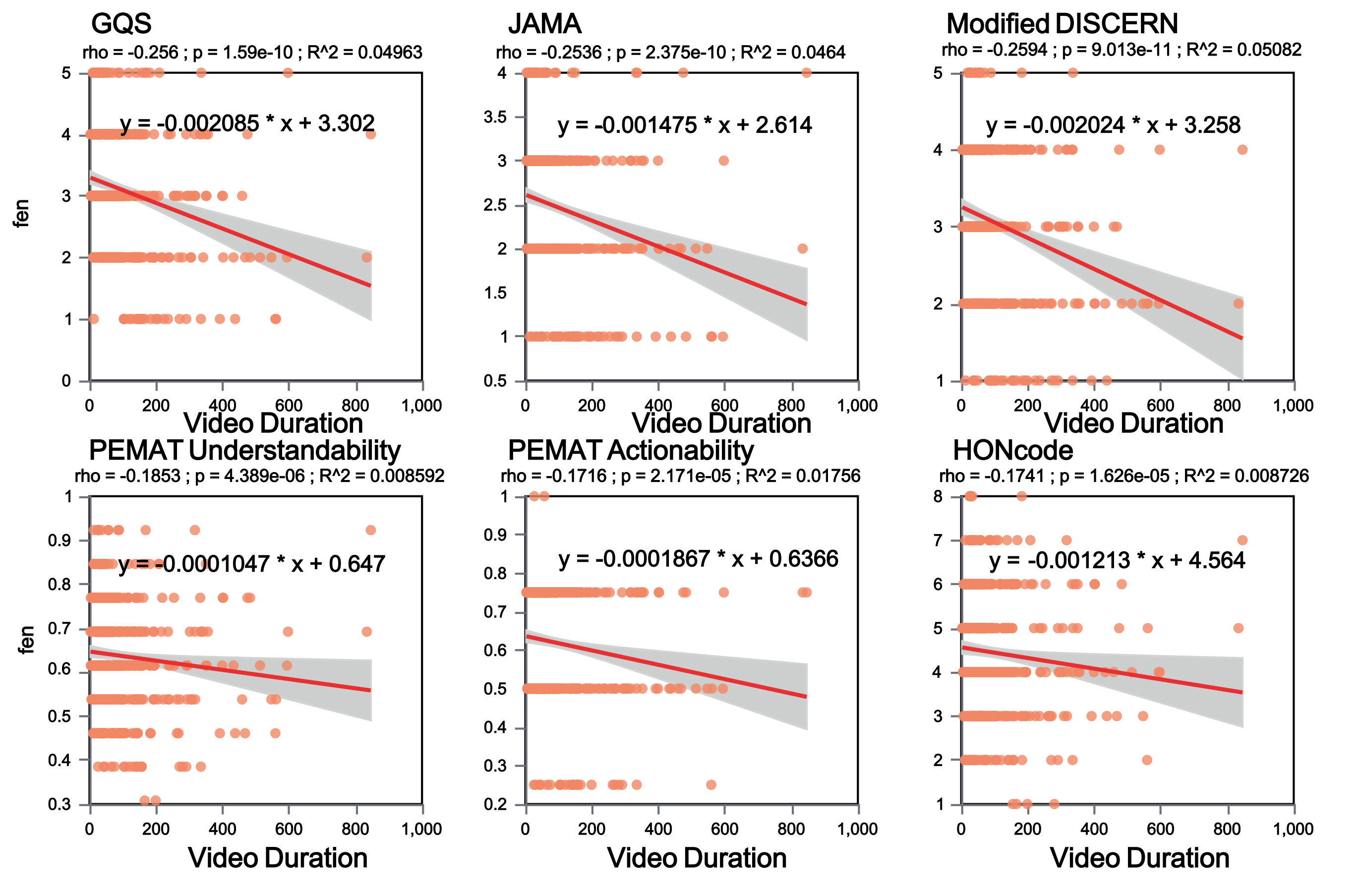


**Supplementary figure 2.** video duration regression analyses with GQS scores, JAMA scores, modified DISCERN scores, PEMAT Understandability scores, Actionability scores, and HONCODE scores.
