# Supplementary material for: Videos in short video sharing platforms as a source of information on bipolar disorder: a cross-sectional content analysis study
Source: Front Public Health. 2025 Oct 28;13:1627885. doi: 10.3389/fpubh.2025.1627885 (PMC12602428; doi:10.3389/fpubh.2025.1627885)
Supplement: Supplementary file 1 [file Data_Sheet_1.zip › supplementary material/Supplementary table 1.docx]

**Supplementary Table 1.** Describe the overall quality score used to assess the quality of informational videos about bipolar disorder.

| Score* | Global Score Description |
| --- | --- |
| 1 score | poor quality, poor traffic, most of the information is missing and therefore not useful to the patient. |
| 2 score | Generally poor quality with poor flow, some information listed but many important topics are missing from the information, making it of limited use to patients. |
| 3 score | medium quality with suboptimal flow, adequate discussion of some key information, but insufficient discussion of other information, making it somewhat helpful to patients. |
| 4 score | Good quality and generally goo flow, most of the relevant information is covered, but some useful topics are missing for patients. |
| 5 score | Excellent quality and excellent flow, very useful for patients. |

*The score ranges from 1 (poor quality) to 5 (excellent flow and quality).
