# Supplementary material for: Videos in short video sharing platforms as a source of information on bipolar disorder: a cross-sectional content analysis study
Source: Front Public Health. 2025 Oct 28;13:1627885. doi: 10.3389/fpubh.2025.1627885 (PMC12602428; doi:10.3389/fpubh.2025.1627885)
Supplement: Supplementary file 1 [file Data_Sheet_1.zip › supplementary material/Supplementary table 2.docx]

**Supplementary Table 2.** Describe the JAMA Benchmark Criteria for assessing the quality of informational videos about bipolar disorder.

| Score* | Score component | |
| --- | --- | --- |
| 1 score | Authorship | Author and contributor credentials and their affiliations should be provided. |
| 1 score | Attribution | Clearly lists all copyright information and states references and sources for content. |
| 1 score | Currency | Initial date of posted content and subsequent updates to content should be provided. |
| 1 score | Disclosure | Conflicts of interest, funding, sponsorship, advertising, support, and video ownership  should be fully disclosed. |

*The criteria of each aspect were scored separately, and 1 point for each criterion with a total score of 4 points.
