# Supplementary material for: Videos in short video sharing platforms as a source of information on bipolar disorder: a cross-sectional content analysis study
Source: Front Public Health. 2025 Oct 28;13:1627885. doi: 10.3389/fpubh.2025.1627885 (PMC12602428; doi:10.3389/fpubh.2025.1627885)
Supplement: Supplementary file 1 [file Data_Sheet_1.zip › supplementary material/Supplementary table 3.docx]

**Supplementary Table 3.** Description of modified DISCERN benchmark criteria for assessing the reliability of informational videos on bipolar disorder.

| Score* | Reliability Score |
| --- | --- |
| 1 score | Is the video clear, concise, and understandable? |
| 1 score | Are reliable sources of information used? (i.e., publication cited, speaker is specialist) |
| 1 score | Is the information presented balanced and unbiased? |
| 1 score | Are additional sources of information listed for patient reference? |
| 1 score | Are areas of uncertainty/controversy mentioned? |

*The criteria of each aspect were scored separately, and 1 point is given for every Yes and 0 points for No.
