# Supplementary material for: Videos in short video sharing platforms as a source of information on bipolar disorder: a cross-sectional content analysis study
Source: Front Public Health. 2025 Oct 28;13:1627885. doi: 10.3389/fpubh.2025.1627885 (PMC12602428; doi:10.3389/fpubh.2025.1627885)
Supplement: Supplementary file 1 [file Data_Sheet_1.zip › supplementary material/Supplementary Table 4.docx]

**Supplementary Table 4.** Description of PEMAT benchmark criteria for assessing the comprehensibility and actionability of informational videos on bipolar disorder.

| PEMAT Understandability |  |
| --- | --- |
| Content | The purpose of the material is expressed very clearly |
| Content | Material does not contain distracting information unrelated to its purpose |
| Diction and style | Use of everyday, easy-to-understand words |
| Diction and style | Medical terms are explained or defined wherever they appear |
| Diction and style | Use of the active voice |
| Digital use | Audiences are not required to make their own calculations |
| Digital use | If figures appear, express them clearly and understandably |
| Structural organisation | Information is presented in a logical and clear sequence |
| Structural organisation | Provide a summary or review of key points |
| Layout | Focus through visual cues (titles, subtitles, bullet points, etc.) |
| Visual aid | Use of visual aids when helpful for understanding |
| Visual aid | Visual aids reinforce rather than distract from content |
| Visual aid | Visual content adapted to the cultural context of the audience |
| **PEMAT Actionability** |  |
| Actionability | Clearly identify at least one action that can be implemented |
| Actionability | Actions are broken down into specific actionable steps |
| Actionability | Provide tools to support action (e.g., checklists) |
| Actionability | Clear instructions on how to use these tools |

Scoring Instructions

1 = ‘Agree’ (fully meets the description)

0 = “Disagree” (does not meet or only partially meets)

NA = ‘Not Applicable’ (this entry is not relevant to the material and is excluded from scoring) )

Calculating Percentages

Comprehensibility Score (%) = (sum of comprehensibility scores ÷ number of comprehensible scorable items) × 100

Operability Score (%) = (sum of operability scores ÷ number of operable scorable items) × 100

Interpretation of results: Usually ≥70% is considered as a good performance for the dimension; values below indicate a need for improvement.
