# Supplementary material for: Videos in short video sharing platforms as a source of information on bipolar disorder: a cross-sectional content analysis study
Source: Front Public Health. 2025 Oct 28;13:1627885. doi: 10.3389/fpubh.2025.1627885 (PMC12602428; doi:10.3389/fpubh.2025.1627885)
Supplement: Supplementary file 1 [file Data_Sheet_1.zip › supplementary material/Supplementary Table 5.docx]

**Supplementary Table 5.** Description of HONCODE benchmark criteria for assessing the reliability and comprehensibility of informational videos on bipolar disorder.

| Information authority | Clearly identifies the author/editor's name, qualifications, and institution  Medical content must be written or reviewed by appropriately qualified professionals |
| --- | --- |
| Complementarity of information | The content of the website is for health education reference only, and is not a substitute for face-to-face consultation between doctors and patients.  should prominently state that‘it is not a substitute for professional medical advice’. |
| Privacy | Respecting User Privacy and Protecting Personal Health Data  Describe how data is collected, stored and used, and obtain consent |
| Sources and dates of information | Data, studies, guides, etc. used must be referenced and hyperlinked  Each article/page should be labelled with the most recent date of update or review |
| Assertion of demonstrability | Any claims about treatment methods, efficacy and statistics must be supported by scientific evidence  Provide references or authoritative guidelines that can be verified. |
| Website transparency | Provide the name, address, and contact information of the owner or operator of the site  Describe the purpose of the site, target audience |
| Funding and Sponsorship Disclosure | Public sources of funding, advertising and sponsorship  Description of business relationships and their potential impact on content |
| Advertising policy | Clearly distinguish between advertising and editorial content (layout or labelling)  Ensure that advertising does not compromise the objectivity of medical content |

According to the number of criteria satisfied by the video, 0~2, 3~5 and 6~8 entries correspond to three levels of video quality: low quality, medium quality and high quality respectively.
